# Who buys nets? Factors associated with ownership and use of purchased mosquito nets in sub-Saharan Africa

**DOI:** 10.1186/s12936-019-3020-7

**Published:** 2019-12-04

**Authors:** Bolanle Olapeju, Ifta Choiriyyah, Kathryn Bertram, Danielle Piccinini, Hunter Harig, Richmond Ato Selby, Matthew Lynch, Hannah Koenker

**Affiliations:** 1 PMI VectorWorks Project, Johns Hopkins Center for Communication Programs, Baltimore, MD USA; 2 Department of Population, Family and Reproductive Health, Johns Hopkins University Bloomberg School of Public Health, Baltimore, USA

**Keywords:** Purchased nets, Insecticide-treated nets, Sub-Saharan Africa, Private-sector, Distribution, Channel

## Abstract

**Background:**

Public sector strategies to promote insecticide-treated net (ITN) access have resulted in increased ITN ownership across sub-Saharan Africa. However, the current status of the private sector distribution channel for nets has not been fully explored. This multi-country study explored the prevalence of net purchases and the characteristics of households that had purchased nets and used such nets in sub-Saharan Africa.

**Methods:**

Data from recent Malaria Indicator Survey (MIS) or Demographic and Health Survey (DHS) in 16 countries were analysed to explore the prevalence of purchased nets. Purchased nets were defined as nets obtained from shops/markets or pharmacies. Additional sub-analysis of factors associated with ownership and use of purchased nets was conducted in seven countries with over 10% of nets reported as purchased. Key outcomes included: prevalence of purchased nets out of all nets, household ownership of a purchased net, and whether a purchased net was used the previous night. Analytical methods included country level tests of association and multivariable logistic regressions.

**Results:**

Among all nets, the proportion of purchased nets in the study countries ranged from 0.8 to 32.7% and most (median = 77%) of these purchased nets were ITNs. Although the private nets are presumed to be from the retail, non-public sector, the prevalence of treated purchased nets suggests that some purchased nets may be “leaked” ITNs from public sector distributions, and thus, may be an informal sector rather than part of the formal “private sector”. Urban, wealthier households as well as those with educated heads were more likely to own purchased nets. Use of such nets was, however, lower in wealthier households. In addition, net use was higher in households owning insufficient nets for their family size, and when the nets were newer than 24 months.

**Conclusion:**

The formal and informal private sector have played a role in bolstering net access rates in some settings. Study findings can help relevant malaria control stakeholders gain insight on the contribution of purchased nets on their overall ITN strategy, identify potential target populations for private sector nets as well as inform the design and distribution of private sector insecticide-treated nets that appeal to their target groups.

## Background

The World Health Organization (WHO) recommends universal insecticide-treated nets (ITN) access [1] for all people at risk of malaria and recommends that countries apply a combination of mass free distributions and continuous distributions through multiple channels [2]. Most countries in sub-Saharan Africa still rely heavily on mass distribution campaigns which accounted for 75% of ITNs distributed between 2014 and 2016 [2]. Between 2010 and 2017, household ownership of at least one ITN increased from 47 to 72% in sub-Saharan Africa, while population access to an ITN increased from 33 to 56% in the same period [3].

Public sector strategies to promote ITN access include mass distribution campaigns (which typically occur every 3 years) and continuous distribution strategies through antenatal care visits, immunization visits, schools and community mechanisms. However, the wear and tear on campaign nets, additional births between campaigns and sub-populations missed by various channels necessitate that additional efforts may be needed to boost or maintain ITN levels. Experience and modelling show that rapid and equitable increases of ITN ownership from low levels can be achieved through free mass campaigns, but a multi-channel continuous distribution strategy is likely needed to sustain universal access at steady rates [4]. Public sector ITN distribution strategies are mainly donor-driven with the United States President’s Malaria Initiative [5] and Global Fund [2, 6] procuring majority of ITNs globally in 2017.

The establishment of a viable private sector market for ITNs is a potential economically sustainable approach to continuous ITN distribution to maintain gains in malaria prevention. A systematic investigation of the current context of purchased nets including their reach and use, in sub-Saharan Africa is crucial to understanding net purchase behaviors within the current market and future opportunities for purchased ITNs. To this end, this study used Malaria Indicator Survey (MIS) or Demographic and Health Survey (DHS) data to systematically address the following questions regarding purchased nets in sub-Saharan Africa:

1. What is the prevalence and insecticide treatment status of purchased nets?
2. What factors are associated with household ownership of at least one purchased net?
3. What factors are associated with a purchased net being used the previous night?
4. Is the use of purchased nets comparable with nets obtained from the public sector?

Insecticide-treated nets distribution strategies have changed significantly in the past 20 years, shifting from subsidized sales through private sector and non-governmental organization channels to an almost completely public sector distribution approach in order to rapidly scale up to universal coverage. The shift to public sector universal coverage campaigns created high awareness regarding ITNs and improved population ITN access and use but likely requires continued donor support. Renewed efforts are needed to strike a sustainable and equitable balance between public and private distribution channels to explicitly target distribution to achieve elimination, reduce operational costs and over-reliance on donor funding. Furthermore, there is an emerging emphasis in ITN distribution strategies on stratification and localized targeting of ITN distribution among specific subgroups to strategically deploy ITN based on data on access and risk of infection. Therefore, it will become important for programmes to effectively leverage private sector ITN sales to complement public sector ITN distribution efforts.

Publications on private sector ITN distribution fall into two eras. Prior to the shift to a “Universal Coverage” paradigm in 2008, several donors supported subsidized sales of nets through social marketing channels or voucher schemes [4, 7–9]. These programmes were not successful in achieving high levels of ITN access, due to limited availability of ITNs [8, 10] and consumer barriers such as distance to sales points and ability to pay [11, 12]. Following 2008, most donors shifted to support for mass campaign distribution in which ITNs were fully subsidized, e.g. free of charge to the recipient. Public sector distribution broadened following work showing the value of continuous distribution channels in sustaining high population access rates, and the addition of continuous distribution to the WHO technical guidance in 2014 [13].

Insecticide-treated nets use is generally high among individuals with access to an ITN [14], but recent literature has not explored whether people are more inclined to use a free versus purchased net/ITN. While it has been argued that charging a price for health goods leads to higher usage rates [15], a systematic review published in 2015 suggests that there is little or no difference in the use of ITNs when they are provided free, compared to providing subsidized ITNs or ITNs offered at full market price [16].

## Methods

This study analysed data from recent (2011 to 2016) MIS or DHS from countries in sub-Saharan Africa that used a two-step source of net question with standardized responses (N = 16). To provide contextual background for the study countries, economic indicators: Gross Domestic Product (GDP) and Human Development Index (HDI) were retrieved from the World Bank https://data.worldbank.org and United Nations Development Program http://hdr.undp.org web sites. Malaria prevalence based on rapid diagnostic testing and population access were obtained from the DHS/MIS reports and timing of mass campaigns from the U.S. President’s Malaria Initiative Malaria Operational Plans (http://www.pmi.gov) corresponding to the year of the survey. The most recent publicly available MIS or DHS datasets from a total of 16 countries were downloaded with permission from the DHS Program web site, http://www.dhsprogram.com. Purchased nets were defined as nets obtained from shops/markets or pharmacies based on response to the DHS/MIS question on the source of each net from the net roster section of the household questionnaire. Of note, the response option does not distinguish between formal and informal markets. Public sector nets were defined as nets from the national mass campaign, government health facility, antenatal care visit, immunization visit, or from schools. Other covariates of interest included household characteristics such as the gender of head of household; age of head of household (< 35, 35–59, 60+ years); household size (1–3; 4–6, 7+ people); household net supply (some but not enough: < 0.5; enough: 0.5 to 1, and too many: > 1 net/person); presence of a currently (at the time of the survey) or recently (within the 12 months of the survey) pregnant woman (yes versus no); presence of a child under 5 years old (yes versus no); and presence of a primary school-aged child, defined as 5 to 14 years of age (yes versus no). Other sociodemographic variables included household wealth quintile (poorest, poorer, middle, richer and richest) based on the standard DHS/MIS wealth index determined by principal component analysis on household assets, residence (urban/rural), and region (subnational administrative divisions for each country).

To assess the prevalence and insecticide treatment status of purchased nets out of all nets in all 16 countries, the proportion of purchased nets was examined by source (pharmacies versus shops/markets) and insecticide treatment status (treated versus untreated) using cross tabulations. Chi-squared tests of association were used to test for differences in proportions and the probability of type I error was set to 0.05. Then at the household level, the proportion of households owning at least one purchased net was compared using cross tabulations. To explore factors associated with household ownership of at least one purchased net, the analysis was restricted to countries with over 10% of nets reported as purchased to have enough sample for the analysis. This included seven countries; Angola, Malawi, Mali, Madagascar, Tanzania, Uganda and Zimbabwe. Multivariable logistic regressions were conducted to explore household level factors associated with ownership of at least one purchased net, controlling for gender, age and education of head of household, household size, presence of a currently/recently pregnant woman, child under 5 years old or primary school aged (5–14 years of age) child, household wealth quintile, urban/rural residence, and region/province.

To investigate factors associated with a purchased net being used the previous night, multivariable logistic regressions were conducted in the seven countries, controlling for the aforementioned variables as well as household net supply, age, and treatment status of the purchased net. Adjusted odds ratios and 95% confidence intervals were presented. For Mali, the gender of head of household was dropped from the regression model due to very low proportion of female headed households (4%). Lowest wealth quintile was also automatically dropped in the logistic regression for Mali due to low percentage of households in the lowest quintile; presumably most of households in the lowest quintile did not own a purchased net.

Finally, the odds of a purchased net being used were compared to nets from the public sector in the seven countries. Multivariable logistic regressions were conducted controlling for gender, age and education of head of household, household size, presence of a currently/recently pregnant woman, child under 5 years old or school aged (5–14 years of age), child wealth quintile, residence, region, household net supply and household ownership of both public and purchased nets, age and treatment status of the net.

Data management and analysis was done using Stata version 14 (Stata Corporation, College Station, Texas, USA) and Excel 2016 (Microsoft Corporation, Seattle, Washington, USA). All country-level analyses used sample weights to adjust for DHS/MIS sample design and individual response rate [17].

## Results

### Description of study countries

Table 1 provides contextual information on the indicators of the economic status and malaria control in the 16 study countries. The per capita GDP and HDI of each country represent the level of socioeconomic status and development, while the malaria prevalence is used as a proxy for malaria risk. The timing of the last mass campaign and the population ITN access provide more background on public sector distribution channels and overall ITN access, respectively.

**Table 1:** Economic status and malaria control indicators of the study countries

Country/data source	GDP per capita (US$)	Human Development Index	Malaria prevalence^a^ (%)	Most recent mass campaign before DHS/MIS	Population with access to ITN (%)
Angola 2015–2016 DHS	3683	0.57	13.5	2013, 2016^b^	19.7
Burkina Faso 2014 MIS	704	0.41	61.4	2014, 2016^c^	71.2
Burundi 2016–2017 DHS	286	0.42	37.9	2014	32.3
Ghana 2016 MIS	1518	0.59	27.9	2014–2015	65.8
Liberia 2016 MIS	455	0.43	44.9	2015	41.5
Madagascar 2016 MIS	402	0.52	5.1	2015	62.1
Malawi 2017 MIS	339	0.48	36.0	2016	63.1
Mali 2015 MIS	750	0.42	32.4	2015	69.5
Nigeria 2015 MIS	1969	0.53	45.1	2013–2015	54.7
Rwanda 2017 MIS	748	0.52	11.8	2016	71.9
Senegal 2016 DHS	953	0.50	0.9	2016	75.7
Sierra Leone 2016 MIS	481	0.41	52.7	2014	37.1
Tanzania 2017 MIS	617	0.50	43.9	2017	82.3
Togo 2017 MIS	580	0.51	30.3	2017	64.6
Zimbabwe 2015 DHS	1033	0.53	n/a	2013–2014	37.2

*DHS* Demographic Health Survey, *GDP* Gross Domestic Product, *MIS* Malaria Indicator Survey, *n/a* not applicable

^a^Measured among children aged 6–59 months using rapid diagnostic testing (RDT)

^b^Angola implemented a phased mass distribution campaign

^c^Burkina Faso’s most recent campaign was after the MIS

Most of the countries had a per capita GDP of less than US $1000. Malaria prevalence was greater than 40% in Burkina Faso (61.4%), Sierra Leone (52.7%), Nigeria (45.1%), Liberia (44.9%), and Tanzania (43.9%). All the countries had a mass distribution campaign within 3 years of the survey. Population access with access to ITN was less than 40% in Angola (19.7%), Burundi (32.3%), Sierra Leone (37.1%), and Zimbabwe (37.2%).

### Prevalence of purchased nets

Table 2 highlights the proportion of households with at least one purchased net across the countries. Ownership of a purchased net ranged from 1.0% in Rwanda to 23.4% in Mali. Stratification by residence and wealth quintile showed that in the majority of countries, urban and wealthier households were significantly more likely to own purchased nets. In most countries, ownership of purchased nets was higher with each increasing wealth quintile.

**Table 2:** Proportion of households with at least one purchased net, by residence and wealth quintile

Country/data source	Overall	Residence		Wealth quintile				
		Urban	Rural	Poorest	Poorer	Middle	Richer	Richest
Angola 2015–2016 DHS	13.1	18.2	5.1*	0.4	0.6	16.9	23.3	19.4*
Burkina Faso 2014 MIS	5.8	11.4	3.4*	3.3	2.4	3.7	4.6	13.1*
Burundi 2016–2017 DHS	5.2	24.5	2.9*	1.1	1.6	2.0	4.3	19.4*
Ghana 2016 MIS	3.8	4.3	3.3	1.7	2.7	3.5	3.8	6.8*
Liberia 2016 MIS	8.2	12.6	2.6*	3.2	3.0	5.8	11.9	8.2*
Madagascar 2016 MIS	11.3	16.9	10.6*	4.2	5.4	7.5	14.2	24.6*
Malawi 2017 MIS	9.9	28.6	6.5*	2.0	3.7	5.5	9.6	29.8*
Mali 2015 MIS	23.4	26.2	22.6	23.3	20.0	22.8	27.8	23.4
Nigeria 2015 MIS	6.2	7.4	5.5*	8.3	5.8	5.5	5.3	6.7*
Rwanda 2014–2015 DHS	1.0	3.1	0.4*	0.0	0.1	0.9	0.4	0.4*
Senegal DHS 2015	8.4	8.7	8.1	6.4	7.4	10.9	8.3	9
Sierra Leone 2016 MIS	4.5	7.0	2.8*	1.8	2.7	2.3	5.1	10.0*
Tanzania 2017 MIS	21.0	40.2	11.2*	6.7	7.1	10.2	24.3	49.5*
Togo MIS 2017	4.7	6.9	3.03*	1.3	1.5	4.1	5.8	9.3*
Uganda 2016 DHS	15.6	30.3	10.5*	6.2	6.7	8.1	39.2	15.6*
Zimbabwe 2015 DHS	12.9	27.8	5.3*	2.1	2.7	7.4	19.2	30.1*

*DHS* Demographic Health Survey, *MIS* Malaria Indicator Survey

*Statistically significant difference by residence or wealth quintile *P* < 0.01

Net-level results showing the different sources of nets across all countries is presented in Additional file 1: Table S1. Across countries (except Rwanda and Senegal), shops/markets were the dominant source of those nets. For example, in Angola, a total of 32.7% of nets were purchased (the sum of 28.9% purchased from shops/markets plus 3.8% from pharmacies). In all countries except Zimbabwe, the majority of purchased nets were reported to be ITNs.

### Factors associated with ownership of purchased nets

The household-level factors associated with ownership of at least one purchased net are presented in Table 3 with regional results presented in Additional file 1: Table S2. Factors significantly associated with ownership of purchased nets included factors related to the household head/members; gender, age, and education of the household head, presence of a child under 5 years old or school aged in the household; and factors related to the household itself, residence; region and wealth quintile. The gender of the household head was significantly associated with ownership of a purchased net in Mali but not in any of the other countries. The age of the household head was a significant predictor of ownership of a purchased net in four countries (Madagascar, Malawi, Uganda and Zimbabwe). In Malawi and Zimbabwe, households whose heads were 35 to 59 years old had 40% higher odds of owning a purchased net compared to similar households whose heads were younger than 35 years old. Conversely, in Madagascar and Uganda households whose heads were older than 35 years old had 30–40% lower odds of owning a purchased net. However, the age of the household head was not a significant predictor of ownership of a purchased net in Angola, Mali, and Tanzania.

**Table 3:** Among countries with over 10% of purchased nets: factors associated with household ownership of at least 1 purchased net

Country/data source	Angola 2015–2016 DHS	Madagascar 2016 MIS	Malawi 2017 MIS	Mali 2015 MIS	Tanzania 2017 MIS	Uganda 2016 DHS	Zimbabwe 2015 DHS
Odds of households owning a purchased net: aOR^a^ (95% CI)							
Gender of head of household (ref: male)							
Female	0.9 (0.8,1.1)	1.1 (0.9,1.4)	1.1 (0.9,1.3)	0.7 (0.5,1.0)	1.1 (0.8,1.3)	1.0 (0.9,1.2)	1.1 (0.9,1.2)
Age of head of household in years (ref: < 35)							
35–59	0.9 (0.7,1.1)	0.8 (0.6,1.0)	*1.4* (*1.1*,*1.6*)	1.1 (0.8,1.5)	0.9 (0.7,1.1)	0.9 (0.7,1.0)	*1.4* (*1.1*,*1.6*)
60+	0.8 (0.5,1.1)	*0.6* (*0.4*,*0.9*)	1.2 (0.9,1.5)	1.2 (0.9,1.6)	1.0 (0.7,1.4)	*0.7* (*0.6*,*0.9*)	1.3 (0.9,1.8)
Education of head of household (ref: none)							
Primary	*1.4* (*1.0*,*1.9*)	n/a^b^	1.0 (0.8,1.3)	n/a^b^	n/a^b^	0.9 (0.7,1.2)	1.3 (0.7,2.5)
≥ Secondary	*1.7* (*1.2*,*2.3*)	n/a^b^	*1.6* (*1.2*,*2.1*)	n/a^b^	n/a^b^	*1.7* (*1.3*,*2.3*)	1.7 (0.9,3.3)
Household size (ref: 1–3)							
4–6	1.1 (0.8,1.4)	1.1 (0.8,1.4)	1.0 (0.8,1.3)	*0.6* (*0.4*,*0.9*)	0.9 (0.7,1.1)	1.0 (0.8,1.2)	0.9 (0.7,1.2)
7+	1.0 (0.7,1.3)	1.0 (0.7,1.4)	1.0 (0.7,1.3)	0.9 (0.6,1.4)	1.2 (0.8,1.8)	1.1 (0.9,1.3)	0.9 (0.7,1.2)
Presence of currently/recently pregnant woman (ref: no)							
Yes	1.2 (1.0,1.4)	1.2 (1.0,1.6)	0.9 (0.8,1.1)	1.1 (0.9,1.4)	1.1 (0.9,1.4)	1.1 (0.9,1.2)	1.1 (0.9,1.3)
Presence of a child under 5 years old (ref: no)							
Yes	1.1 (0.9,1.2)	*1.2* (*1.0*,*1.4*)	0.7 (0.6,0.8)	0.8 (0.6,1.1)	0.8 (0.6,1.0)	0.9 (0.8,1.0)	1.3 (1.1,1.6)
Presence of a school aged child (ref: no)							
Yes	0.9 (0.7,1.1)	0.9 (0.7,1.1)	*0.8* (*0.7*,*1.0*)	1.0 (0.8,1.4)	0.8 (0.6,1.0)	*0.7* (*0.6*,*0.9*)	1.2 (1.0,1.4)
Residence (ref: rural)							
Urban	1.5 (1.1,2.1)	0.9 (0.7,1.3)	*1.6* (*1.3*,*2.0*)	0.8 (0.5,1.2)	*1.6* (*1.2*,*2.0*)	1.2 (1.0,1.5)	*2.1* (*1.5*,*3.1*)
Wealth quintile (ref: poorest)							
Poorer	*1.7* (*1.2*,*2.4*)	1.2 (0.8,2.0)	*1.5* (*1.1*,*2.0*)	0.9 (0.5,1.5)	*1.2* (*0.8*,*1.6*)	*1.3* (*1.1*,*1.7*)	*1.5* (*0.9*,*2.6*)
Middle	*3.6* (*2.3*,*5.4*)	1.4 (0.9,2.1)	*2.4* (*1.7*,*3.2*)	1.1 (0.7,1.8)	*1.6* (*1.1*,*2.3*)	*2.0* (*1.5*,*2.5*)	*3.7* (*2.2*,*6.1*)
Richer	*4.47* (*2.73*,*7.31*)	*3.33* (*2.15*,*5.16*)	*3.83* (*2.80*,*5.23*)	1.50 (0.94,2.40)	*4.23* (*2.87*,*6.22*)	*2.80* (*2.13*,*3.69*)	*6.58* (*3.81*,*11.34*)
Richest	*3.3* (*1.9*,*5.5*)	*7.9* (*5.0*,*13.0*)	*10.5* (*7.7*,*14.4*)	*3.3* (*1.9*,*5.7*)	*10.9* (*7.3*,*16.2*)	*8.6* (*6.4*,*11.5*)	*8.5* (*4.7*,*15.4*)
N of households with private nets	1705	1058	2685	970	1731	2914	1654

Italicized values are statistically significant with *P* < 0.05

*CI* confidence interval, *N*- number, *n/a* not applicable, *aOR* adjusted odds ratio, *ref* reference

^a^Adjusted for gender, age and education of head of household, household size, presence of currently/recently pregnant woman, child under 5 years old, or school aged child in the household, residence, wealth quintile and region (shown in Additional file 1: Table S2)

^b^There was no variable for education level of head of household in the dataset

Household size was only significantly associated with ownership of a purchased net in Mali where households with 4 to 6 members had 40% lower odds of owning a purchased net compared to similar households with less than four members. The education level of the head of the household was significantly associated with ownership of a purchased net in many countries. In Angola, households whose head had a primary (adjusted odds ratio (aOR): 1.4) or secondary (aOR: 1.7) education had higher odds to own a purchased net than households whose heads had no education. In Malawi and in Uganda, households that had heads with a secondary or greater education had about 60–70% higher odds of owning a purchased net. The surveys in Madagascar, Mali, and Tanzania did not ask about the education level of the head of the household but instead asked about the mother’s level of education.

While the presence of a pregnant woman in the household was not significantly associated with ownership of purchased net across all countries, the presence of children under 5 years old and school-aged children was significantly associated with ownership of purchased net in some countries. In Malawi, households with children under 5 years had 30% lower odds of owning a purchased net compared to households without children under 5 years but in Zimbabwe on the other hand, households with children under 5 years had 30% higher odds of owning purchased net. In Malawi (aOR: 0.8) and Uganda (aOR: 0.7), households with school-aged children had lower odds of owning a purchased net than the households who did not have school-aged children. Household residence in an urban versus rural area was significantly associated with ownership of a purchased net in many countries. Specifically, households in urban areas had over 50% higher odds of owning a purchased net in four countries; Angola (aOR: 1.5), Malawi (aOR: 1.6), Tanzania (aOR: 1.6), and Zimbabwe (aOR: 2.1). In Madagascar, Mali, and Uganda, ownership of purchased nets was not significantly different across urban and rural households. Specifically, for Madagascar, the confidence interval was 0.97 and 1.51.

Wealth quintile was a major predicator of ownership of a purchased net. In all countries, households in the higher wealth quintiles had higher odds to own a purchased net than poorer households. In Madagascar, Malawi, Uganda and Zimbabwe, the odds of owning a purchased net was significantly higher with each increasing wealth quintile.

### Factors associated with use of purchased nets

Table 4 presents the odds of a purchased net being reported as used the previous night from the net roster, adjusted for gender of head of household, age of head of household, education of head of household, household net supply, household size, presence of a currently/recently pregnant woman, presence of a child under 5 years old, residence, wealth quintile, and region. Regional differences are not shown in Table 4, but in Additional file 1: Table S3. The gender, age, and education of the household head as well as the presence of public-sector nets or school aged children in the household were not significantly associated with a purchased net being used the previous night across all countries. Factors significantly associated with use of purchased nets were seen at the net level; supply of nets in the household, treatment status and age of the purchased nets; household member level; presence of a pregnant woman or child under 5 years old in the household; and household level; residence and wealth quintile.

**Table 4:** Among countries with over 10% purchased nets: factors associated with a purchased net being used the previous night

Country/data source	Angola 2015–2016 DHS	Madagascar 2016 MIS	Malawi 2017 MIS	Mali 2015 MIS	Tanzania 2017 MIS	Uganda 2016 DHS	Zimbabwe 2015 DHS
Adjusted odds of a purchased net being used the previous night: aOR^a^ (95% CI)							
Gender of head of household (ref: male)							
Female	0.8 (0.5,1.2)	1.7 (1.0,2.9)	1.1 (0.8,1.5)	n/a^a^	1.14 (0.8,1.7)	1.12 (0.8,1.5)	1.1 (0.8,1.5)
Age of head of household in years (ref: < 35)							
35–59	1.1 (0.7,1.6)	1.3 (0.8,2.1)	0.9 (0.7,1.3)	0.7 (0.1,3.6)	1.0 (0.7,1.3)	1.1 (0.9,1.5)	1.4 (1.0,2.0)
60+	0.8 (0.3,1.7)	1.2 (0.5, 2.9)	1.4 (0.8,2.5)	1.2 (0.2,7.0)	0.9 (0.6,1.4)	1.2 (0.8,1.8)	0.7 (0.4,1.3)
Education of head of household (ref: none)							
Primary	1.0 (0.5,2.0)	n/a^c^	1.2 (0.7,2.0)	n/a^c^	n/a^c^	0.7 (0.4,1.5)	1.7 (0.3,8.8)
≥ Secondary	1.4 (0.7,2.8)	n/a^c^	1.2 (0.7,2.0)	n/a^c^	n/a^c^	0.7 (0.4,1.5)	0.8 (0.2,4.5)
Household net supply (nets/person; ref: some but not enough (< 0.5 net/person))							
Enough (0.5–1 net/person)	*0.6* (*0.4*,*1.0*)	*0.5* (*0.3*,*0.9*)	0.7 (0.5,1.1)	1.6 (0.7,3.8)	0.8 (0.5,1.3)	*0.5* (*0.4*,*0.8*)	1.2 (0.7,2.1)
Too many (> 1 net/person)	*0.1* (*0.0*,*0.3*)	*0.2* (*0.1*,*0.8*)	*0.2* (*0.1*,*0.4*)	n/a^b^	*0.2* (*0.1*,*0.4*)	*0.1* (*0.1*,*0.2*)	0.7 (0.2,2.2)
Insecticide-treatment status of nets (ref: non-ITN)							
ITN	1.2 (0.8,1.9)	1.4 (0.8,2.3)	*1.7* (*1.3*,*2.3*)	1.1 (0.3,3.9)	1.2 (0.8,1.9)	*2.3* (*1.5*,*3.3*)	1.0 (0.7,1.5)
Age of the net (ref: ≤ 6 months)							
7–12 months	1.4 (0.9,2.2)	1.0 (0.5,1.9)	0.8 (0.6,1.2)	3.9 (1.0,14.8)	0.9 (0.6,1.4)	1.2 (0.9,1.6)	1.0 (0.7,1.6)
13–24	*2.0* (*1.1*,*3.4*)	0.7 (0.3,1.5)	0.7 (0.5,1.1)	3.6 (0.7,17.7)	0.9 (0.6,1.5)	0.8 (0.6,1.1)	0.8 (0.5,1.3)
≥ 25 months	1.0 (0.6,1.6)	*0.5* (*0.3*,*0.8*)	0.7 (0.5,1.1)	1.4 (0.5,4.0)	0.8 (0.5,1.2)	*0.6* (*0.4*,*0.9*)	*0.6* (*0.4*,*0.9*)
Presence of public sector net in the household (ref: no)							
Yes	0.9 (0.5,1.6)	0.8 (0.4,1.5)	1.1 (0.8,1.5)	0.6 (0.2,2.2)	0.8 (0.6,1.1)	1.0 (0.7,1.3)	0.9 (0.6,1.4)
Presence of currently/recently pregnant woman (ref: no)							
Yes	1.5 (1.0,2.1)	1.1 (0.6,1.8)	1.0 (0.7,1.5)	0.9 (0.3,2.6)	1.5 (1.0,2.2)	0.8 (0.7,1.1)	1.3 (0.9,1.9)
Presence of a child under 5 years old (ref: no)							
Yes	1.0 (0.7,1.4)	0.7 (0.4,1.1)	0.8 (0.6,1.2)	1.0 (0.3,3.5)	1.2 (0.9,1.7)	1.1 (0.8,1.5)	1.0 (0.7,1.5)
Presence of a school aged child (ref: no)							
Yes	0.8 (0.5,1.2)	1.1 (0.7,1.7)	1.2 (0.9,1.6)	2.6 (0.6,10.8)	1.4 (0.9,2.2)	*1.3* (*1.1*,*1.7*)	0.7 (0.5,1.0)
Residence (ref: rural)							
Urban	1.0 (0.5,2.0)	0.7 (0.4,1.4)	*1.7* (*1.3*,*2.3*)	0.8 (0.3,1.9)	1.4 (1.0,2.1)	1.3 (0.9,1.8)	1.8 (0.7,4.2)
Wealth quintile (ref: poorest)							
Poorer	*2.3* (*1.1*,*4.9*)	0.9 (0.3,2.4)	0.8 (0.3,1.8)	–	*3.0* (*1.4*,*6.5*)	0.7 (0.4,1.3)	2.3 (0.5,10.3)
Middle	*3.6* (*1.4*,*9.0*)	1.5 (0.5,4.3)	1.3 (0.6,2.9)	0.4 (0.1,2.6)	*2.4* (*1.1*,*5.3*)	*0.5* (*0.2*,*0.9*)	1.8 (0.4,7.8)
Richer	2.5 (0.9,6.4)	1.2 (0.5,3.0)	1.1 (0.5,2.2)	0.3 (0.1,2.1)	*3.1* (*1.7*,*5.9*)	*0.5* (*0.3*,*0.8*)	1.0 (0.2,4.7)
Richest	1.6 (0.6,4.1)	1.2 (0.5,3.2)	1.0 (0.5,2.1)	0.3 (0.1,1.3)	*2.8* (*1.5*,*5.2*)	*0.5* (*0.3*,*0.9*)	1.0 (0.2,4.7)
N of nets from private source	2641	1364	4131	1871	2634	5105	2415

Italicized values are statistically significant with *P* < 0.05

*CI* confidence interval, *HH* households, *ITN* insecticide-treated nets, *N* number, *n/a* not applicable, *aOR* adjusted odds ratio, *ref* reference

^a^Adjusted for gender, age and education of head of household, household net supply, insecticide treatment status and age of nets, presence of public-sector net in the household, presence of currently/recently pregnant woman, child under 5 years old, or school aged child in the household, residence, wealth quintile and region (shown in Additional file 1: Table S3)

^b^The variable was dropped in the logistic regression due to small sample size or there were no observations

^c^There was no variable for education level of head of household in the dataset

Household supply of nets was a significant predictor of purchased net use in many countries. In Angola, Madagascar, and Uganda, purchased nets in households with not enough nets (< 0.5 nets per person) were more likely to be used the previous night than purchased nets in households with not enough (0.5 to 1 net per person). Similarly, in Angola, Madagascar, Malawi, Tanzania, and Uganda, purchased nets in the households with more than enough nets (> 1 net per person) had lower odds of being used the previous night. Of note, among the countries, the aOR of purchased nets being used in households with more than enough nets ranged from 0.1 in Angola to 0.2 in both Madagascar and Tanzania. In Mali and Zimbabwe, the household net supply was not significantly associated with a purchased net being used the previous night.

The treatment status of the purchased net was a significant predictor of use the previous night in two countries. Insecticide-treated purchased nets had higher odds of being used the previous night compared to untreated purchased nets in Malawi (aOR: 1.7) and Uganda (aOR: 2.3). Similarly, the age of purchased nets was a significant predictor of use the previous night in a few countries. In Angola, nets that were 13 to 24 months old had two times the odds of being used the previous night than nets less than 7 months old. In Madagascar, Uganda, and Zimbabwe, nets that were more than 24 months old had 40–50% lower odds of being used.

The presence of pregnant women in the household was significantly associated with a purchased net being used the previous night in Angola only (aOR: 1.5). The presence of school aged children in the household was associated with 30–40% higher odds of using purchased nets in Tanzania and Uganda but 30% lower odds in Zimbabwe. Purchased nets in urban households had 70% higher odds of being used the previous night compared to rural households in Malawi. Wealth quintile was significantly associated with a purchased net being used the previous night in some countries. In Angola, purchased nets in the poorer and middle households had higher odds of being used than purchased nets in the poorest households. While in Tanzania, purchased nets in households other than the poorest households had higher odds of being used. In Uganda, purchased nets in the middle, richer, and richest households had lower odds of being used. Of note, in Mali, none of the factors included in the model were significantly associated with a purchased net being used the previous night.

Across all the countries included in the analysis, the odds of a purchased net being used was either significantly higher or comparable to public sector nets and this is presented in Table 5. Specifically, in Angola (aOR: 1.5) and Tanzania (aOR: 1.7), the odds of purchased nets being used the previous night was significantly higher than that of public sector nets. However, in Madagascar, Malawi, Mali, Uganda and Zimbabwe, there was no significant difference in the odds of purchased or public sector nets being used.

**Table 5:** Among countries with over 10% purchased nets: adjusted odds of net use by source

Country/data source	Angola 2015–2016 DHS	Madagascar 2016 MIS	Malawi 2017 MIS	Mali 2015 MIS	Tanzania 2017 MIS	Uganda 2016 DHS	Zimbabwe 2015 DHS
Adjusted odds of a purchased net being used the previous night, compared to other sources: aOR^a^ (95% CI)							
Source of net (ref: public-sector)							
Private	*1.5* (*1.2*,*2.0*)	*1.4* (*1.1*,*1.9*)	1.0 (0.8,1.2)	1.5 (0.7,3.2)	*1.7* (*1.3*,*2.3*)	1.2 (1.0,1.6)	1.2 (0.9,1.6)
Other	1.1 (0.6,1.8)	1.1 (0.9,1.3)	0.9 (0.7,1.1)	0.8 (0.5,1.2)	0.9 (0.5,1.6)	0.8 (0.6,1.1)	1.0 (0.7,1.3)
N of nets from any source	10,653	18,593	33,407	15,198	18,088	37,657	12,442

Italicized values are statistically significant with *P* < 0.05

*CI* confidence interval, *N* number, *aOR* adjusted odds ratio, *ref* reference

^a^Adjusted for gender, age and education of head of household, household net supply, insecticide treatment status and age of nets, presence of public-sector net in the household, presence of currently/recently pregnant woman, child under 5 years old, or school aged child in the household, residence, wealth quintile and region

## Discussion

This study explored ownership and use of purchased nets and found that in seven countries (Angola, Madagascar, Malawi, Mali, Tanzania, Uganda and Zimbabwe) at least 10% of all nets in households had been purchased. These higher levels of purchased net ownership may be partly attributed to a low overall ownership of public sector ITNs. It is not possible, using these data, to determine which nets may be ITNs which have “leaked” from public sector distributions, including mass distribution campaigns in the same country or from neighbouring countries. As their free public sector nets wear out, some households appear to be turning to the private sector to replace those nets. The majority of purchased nets were insecticide-treated, with the exception of Zimbabwe.

Urban, wealthier households and households with educated heads were more likely to own purchased nets, corroborating other studies [11, 18–20]. Educated household heads may have a greater understanding of the threats of malaria, perceive the need to use mosquito nets to prevent infection, have stronger preferences for particular net characteristics [21], and a have adequate resources to procure purchased nets. A 2018 ITN market analysis in Ghana found that middle-class respondents preferred (and were willing to pay for) nets that were insecticide-treated, larger and with other features the public sector nets lacked [22, 23]; a similar demand for treated nets with preferred characteristics such as shape, size and colour was observed in a Tanzania study [24]. Other reasons households might purchase nets include major gaps in access (for example, the entire household was missed during the previous mass campaign) or minor gaps in access (for example, school aged children might no longer want to share a net with other household members). The low proportion of households that owned both private and public nets in many countries suggests that households purchase nets when they have no nets more often than to fill gaps within a household.

By far the biggest factor in use of purchased nets was the overall availability of nets within the household; in households with not enough nets, a purchased net was up to ten times more likely to be used. Newer nets, more likely to be in better condition, were also more likely to have been used. This study observed that the odds of purchased nets being used was comparable or higher than that of public sector nets, suggesting that these nets are purchased to fill specific gaps in access or to meet preferences, and corroborating findings from a systematic review [16].

While efforts are needed to clarify the leakage of campaign nets into the private sector, the study findings suggest that net purchase behaviors are not uncommon and there is a demand for purchasable nets in some settings. Thus, net purchases supplement mass distributions and provide households with options for replacing or increasing the number of nets they own with products that best fit their needs. Improving economic conditions in many African countries have increased the pool of potential consumers, particularly in urban areas, and experience with a variety of types of public-sector nets has led many consumers to have preferences for textile, shape, colour, and size [21]. Poorer households are less likely to purchase nets, but demand is not zero [12]; a recent study indicated that poorer households in rural areas showed a higher likelihood of buying a net compared to urban households [24]. Results of this study may provide an impetus for the private sector to promote retail sales of insecticide-treated nets (versus untreated nets) that may appeal to their audience’s preferred features including shape, size, texture, and aesthetics.

An advantage of the recent donor-led effort to limit options in size, colour, and shape of public-sector ITNs is that a market niche has been opened for ITNs with different or more desirable characteristics, which may finally provide differentiation in the ITN market, and separate the public-sector free nets from commercially-available products. Even in settings that have a weak private sector market for ITNs, there is a potential to catalyse the private sector market. A 2007 review reported that cost-sharing schemes incorporating modest targeted subsidies have promoted ITN access and reduced malaria transmission and could represent an important option for national programmes lacking adequate financing to fully subsidize comprehensive ITN coverage [25].

Leveraging the private sector to bolster overall access and reduce the expenditure burden on national programmes is a complex process yet to be tested in the era of universal coverage, or to receive WHO formal operational guidance, let alone political support. However, knowing where purchased nets are more likely to be found can help inform distribution and marketing strategies. The likelihood of wealthier households to purchase nets may complement the likelihood of poorer households to own public sector nets, and potentially offer opportunities for more efficient allocation of public sector funds for malaria prevention through targeting free distribution of public sector nets. The lower risk profile of many wealthier, urban households may provide limited public health returns on increased public sector investment targeted at increasing access and/or use in those households.

This study acknowledges some limitations. First, given the prevalence of purchased ITNs, it is likely that in some countries, some purchased nets were in fact ‘*leaked*’ public sector nets (originally procured by the public sector for free distribution but now sold in the private sector). This study could not differentiate leaked public sector from private sector nets as response options do not distinguish between formal and informal markets. Research conducted in Ghana, Nigeria and Tanzania show that leaked nets accounted for 47%, 23% and 11% of the market share with considerable cross-country trafficking of leaked nets (Kilian et al., in preparation). Second, some respondents might not accurately remember where the nets came from, though people are more likely to remember if they paid for a product. Third, the study analysis is limited to the examination of household level factors influencing private nets ownership and usage. Thus, this study could not explore important individual level factors such as demographic profile of the specific individual who purchased the net and individual perceptions regarding ITN use or malaria prevention. Finally, the data is also cross sectional and thus does not infer causality or acknowledge temporal changes in net ownership or use. For example, households might have owned purchased nets and discarded or given them away prior to the survey.

## Conclusion

The formal and informal private sector have played a role in bolstering ITN access rates in some settings. This study demonstrated than urban, wealthier households and households with educated heads were more likely to own purchased nets. Use of purchased nets was associated with the overall availability of all nets within the household as well as the age of the purchased nets. In addition, use of purchased nets was comparable or higher than that of public sector nets. Study findings can help relevant malaria control stakeholders gain insight on the contribution of purchased nets on their overall ITN strategy as well as inform the design and implementation of market shaping strategies to shift target populations such as urban, middle/upper class households to purchase retail ITNs. Results of this study may provide an impetus for the private sector to promote the purchase of ITNs (versus untreated nets) that may appeal to their audience’s preferred features including shape, size, texture, and aesthetics.

## Supplementary information

**Additional file 1: Table S1.** Proportion of nets from all sources; and sub-classification of purchased nets by source and insecticide-treatment status. **Table S2.** Among countries with over 10% of nets from the private sector: factors associated with household ownership of at least 1 private-sector net: region. **Table S3.** Among countries with over 10% of nets from the private sector: factors associated with a purchased net being used the previous night: region.

## Data Availability

The datasets analysed during the current study are available from the DHS Program web site, http://www.dhsprogram.com.

## Abbreviations

aOR: adjusted odds ratio
ANC: antenatal care
CI: confidence interval
DHS: Demographic Health Survey
EPI: Expanded Programme on Immunization
GDP: Gross Domestic Product
HDI: Human Development Index
HH: households
ITN: insecticide-treated nets
MIS: Malaria Indicator Survey
RDT: rapid diagnostic testing
WHO: World Health Organization
