# Supplementary material for: Who buys nets? Factors associated with ownership and use of purchased mosquito nets in sub-Saharan Africa
Source: Malar J. 2019 Dec 4;18:401. doi: 10.1186/s12936-019-3020-7 (PMC6894199; doi:10.1186/s12936-019-3020-7)
Supplement: Supplementary file 1 — Additional file 1: Table S1. Proportion of nets from all sources; and sub-classification of purchased nets by source and insecticide-treatment status. Table S2. Among countries with over 10% of nets from the private sector: factors associated with household ownership of at least 1 private-sector net: region. Table S3. Among countries with over 10% of nets from the private sector: factors associated with a purchased net being used the previous night: region. [file 12936_2019_3020_MOESM1_ESM.docx]

**Manuscript Title: Who Buys Nets? Factors associated with ownership and use of purchased mosquito nets in sub-Saharan Africa**

**List of Additional Tables and Figures**

**Additional Table S1: Proportion of nets from all sources; and sub-classification of purchased nets by source and insecticide-treatment status**

| **Country/**  **Data source** | **Sources of all nets^1^ (%)** | | | **Specific sources among purchased^2^ nets (%)** | | **Insecticide-treatment status of purchased nets (%)** | |
| --- | --- | --- | --- | --- | --- | --- | --- |
|  | **Public sector^2^** | **Other source** | **Purchased^2^** | **Pharmacy** | **Shops/**  **Market** | **Treated net** | **Untreated net** |
| Angola 2015-16 DHS | 62.6 | 4.8 | 32.7 | 3.8 | 28.9 | 23.7 | 9.0 |
| Burkina Faso 2014 MIS | 93.0 | 3.1 | 3.9 | 0.8 | 3.1 | 3.2 | 0.7 |
| Burundi 2016-17 DHS | 88.9 | 2.4 | 8.8 | 0.2 | 8.6 | 7.6 | 1.2 |
| Ghana 2016 MIS | 92.0 | 5.0 | 3.1 | 0.7 | 2.3 | 2.4 | 0.7 |
| Liberia 2016 MIS | 87.2 | 3.5 | 9.4 | 0.0 | 9.4 | 8.8 | 0.6 |
| Madagascar 2016 MIS | 85.3 | 5.6 | 9.1 | 0.1 | 9.0 | 6.1 | 3.0 |
| Malawi 2017 MIS | 81.3 | 5.1 | 13.7 | 0.1 | 13.6 | 10.2 | 3.5 |
| Mali 2015 MIS | 78.1 | 9.3 | 12.6 | 0.4 | 12.2 | 10.2 | 2.4 |
| Nigeria 2015 MIS | 91.4 | 1.9 | 6.7 | 0.3 | 6.4 | 5.8 | 0.9 |
| Rwanda 2017 MIS | 97.9 | 1.3 | 0.8 | 0.5 | 0.3 | 0.5 | 0.3 |
| Senegal 2016 DHS | 92.6 | 3.5 | 3.9 | 2.3 | 1.6 | 3.5 | 0.4 |
| Sierra Leone 2016 MIS | 92.0 | 2.6 | 5.4 | 0.2 | 5.2 | 4.9 | 0.5 |
| Tanzania 2015-16 DHS | 66.5 | 5.5 | 28.1 | 0.2 | 27.9 | 18 | 10.1 |
| Tanzania 2017 MIS | 82.5 | 0.9 | 16.6 | 0.4 | 16.2 | 9.7 | 6.9 |
| Togo 2017 MIS | 93.9 | 3.6 | 2.5 | 1.1 | 1.4 | 1.8 | 0.7 |
| Uganda 2016 DHS | 84.0 | 2.0 | 14.1 | 0.5 | 13.5 | 11.8 | 2.3 |
| Zimbabwe 2015 DHS | 73.2 | 11.2 | 15.7 | 0.3 | 15.4 | 6.5 | 9.2 |
| Abbreviations: DHS: Demographic Health Survey, MIS: Malaria Indicator Survey  ^1^May be over 100% due to rounding. ^2^ Purchased nets were defined as nets obtained from shops/markets or pharmacies. ^3^ Public sector nets were defined as nets from the national mass campaign, government health facility, antenatal care visit, immunization visit, or from school. | | | | | | | |

**Additional Table S2: Among countries with over 10% of nets from the private sector: Logistic regression of the factors associated with household ownership of at least 1 private-sector net: per region**

| **Angola 2015-16 DHS** | | **Madagascar 2016 MIS** | | **Malawi 2017 MIS** | | **Mali 2015 MIS** | | **Tanzania 2017 MIS** | | **Uganda 2016 DHS** | | **Zimbabwe 2015 DHS** | |
| --- | --- | --- | --- | --- | --- | --- | --- | --- | --- | --- | --- | --- | --- |
| **Adjusted odds of households owning a purchased net by region: aOR^1^ (95% CI)** | | | | | | | | | | | | | |
| Cabinda | ref | Analamanga | ref | Northern region | ref | Kayes | ref | Dodoma | ref | Kampala | ref | Manicaland | ref |
| Zaire | 0.5 (0.3,0.7) | Vakinankaratra | 0.04 (0.0,0.2) | Central region | 0.8 (0.6,1.1) | Koulikoro | 0.6 (0.3,1.0) | Arusha | 0.5 (0.2,1.2) | Central 1 | 1.0 (0.8,1.4) | Mahonaland Central | 1.3 (0.8,2.1) |
| Uige | 1.1 (0.6,2.0) | Itasy | 1.0 (0.5,2.2) | Southern region | 1.3 (1.1,1.64) | Sikasso | 0.6 (0.3,1.1) | Kilimanjaro | 0.5 (0.2,1.4) | Central 2 | 0.9 (0.6,1.3) | Mashonaland East | 1.8 (1.1,3.0) |
| Luanda | 0.9 (0.6,1.2) | Bongolova | 0.0 (0.0,0.1) |  |  | Segou | 2.5 (1.4,4.5) | Tanga | 0.6 (0.3,1.4) | Busoga | 1.2 (0.8,1.7) | Mashonaland West | 1.8 (1.2,2.7) |
| Cuanza Norte | 1.0 (0.7,1.5) | Haute Matsiatra | 0.6 (0.4,1.0) |  |  | Mopti | 2.6 (1.5,4.5) | Morogoro | 1.1 (0.4,2.6) | Bukedi | 0.9 (0.6,1.3) | Matabeleland North | 0.7 (0.3,1.2) |
| Cuanza Sul | 0.8 (0.5,1.5) | Amoron I Mania | 0.3 (0.2,0.7) |  |  | Bamako | 0.7 (0.4,1.2) | Pwani | 1.5 (0.6,3.5) | Bugishu | 0.6 (0.4,1.0) | Matabeleland South | 2.0 (1.1,3.4) |
| Malanje | 0.5 (0.3,0.7) | Vatovavy Fitovinany | 0.6 (0.3,1.2) |  |  |  |  | Dar Es Salaam | 1.2 (0.6,2.7) | Teso | 6.5 (4.5,9.3) | Midlands | 2.1 (1.3,3.2) |
| Lunda Norte | 0.2 (0.1,0.3) | Ihorombe | 0.2 (0.1,0.5) |  |  |  |  | Lindi | 3.5 (1.6,7.7) | Karamoja | 0.6 (0.3,1.2) | Masvingo | 1.5 (0.9,2.3) |
| Benguenla | 1.2 (0.8,1.7) | Atsimo Atsinanana | 0.4 (0.2,0.8) |  |  |  |  | Mtwara | 4.5 (2.1,9.9) | Lango | 0.8 (0.6,1.1) | Harare | 1.3 (0.9,1.9) |
| Huambo | 0.6 (0.4,1.0) | Atsinanana | 0.5 (0.3,0.9) |  |  |  |  | Ruvuma | 1.1 (0.4,2.8) | Acholi | 1.1 (0.8,1.6) | Bulawayo | 2.8 (1.9,4.2) |
| Bie | 0.2 (0.1,0.3) | Analanjirofo | 2.9 (1.6,5.1) |  |  |  |  | Iringa | 0.3 (0.1,1.0) | West Nile | 1.5 (1.0,2.5) |  |  |
| Moxico | 1.0 (0.6,1.9) | Alaotra Mangoro | 0.3 (0.2,0.4) |  |  |  |  | Mbeya | 0.7 (0.3,1.7) | Bunyoro | 0.6 (0.4,0.9) |  |  |
| Luanda Cubango | 1.5 (1.0,2.1) | Boeny | 0.4 (0.2,0.7) |  |  |  |  | Singida | 0.4 (0.2,1.1) | Toor | 0.4 (0.3,0.6) |  |  |
| Namibe | 0.4 (0.2,0.5) | Sofia | 1.0 (0.6,1.7) |  |  |  |  | Tabora | 1.1 (0.5,2.5) | Ankole | 0.5 (0.4,0.7) |  |  |
| Huila | 0.9 (0.6,1.3) | Betsiboka | 0.3 (0.2,0.7) |  |  |  |  | Rukwa | 1.0 (0.4,2.7) | Kigezi | 0.4 (0.3,0.6) |  |  |
| Cunene | 1.2 (0.8,1.8) | Melaky | 0.6 (0.3,0.9) |  |  |  |  | Kigoma | 0.9 (0.3,2.4) |  |  |  |  |
| Lunda Sul | 0.4 (0.3,0.7) | Atsimo Andrefana | 0.2 (0.0,0.7) |  |  |  |  | Shinyanga | 1.4 (0.6,3.1) |  |  |  |  |
| Bengo | 0.1 (0.1,0.2) | Androy | 0.2 (0.1,0.4) |  |  |  |  | Kagera | 0.6 (0.2,1.6) |  |  |  |  |
|  |  | Anosy | 0.2 (0.1,0.5) |  |  |  |  | Mwanza | 0.8 (0.4,1.8) |  |  |  |  |
|  |  | Menabe | 0.9 (0.4,1.9) |  |  |  |  | Mara | 1.3 (0.6,2.9) |  |  |  |  |
|  |  | Diana | 0.7 (0.4,1.3) |  |  |  |  | Manyara | 0.4 (0.2,1.1) |  |  |  |  |
|  |  | Sava | 2.4 (1.3,4.4) |  |  |  |  | Njombe | 0.2 (0.0,1.1) |  |  |  |  |
|  |  |  |  |  |  |  |  | Katavi | 1.1 (0.5,2.5) |  |  |  |  |
|  |  |  |  |  |  |  |  | Simiyu | 0.8 (0.3,1.9) |  |  |  |  |
|  |  |  |  |  |  |  |  | Geita | 0.4 (0.2,1.1) |  |  |  |  |
|  |  |  |  |  |  |  |  | Songwe | 0.3 (0.2,0.7) |  |  |  |  |
|  |  |  |  |  |  |  |  | Kaskazihi Unguja | 0.2 (0.1,0.7) |  |  |  |  |
|  |  |  |  |  |  |  |  | Kusini Unguja | 0.1 (0.0,0.5) |  |  |  |  |
|  |  |  |  |  |  |  |  | Mjini Magharibi | 0.4 (0.2,0.8) |  |  |  |  |
|  |  |  |  |  |  |  |  | Kaskazini Pemba | 0.2 (0.1,0.7) |  |  |  |  |
|  |  |  |  |  |  |  |  | Kusini Pemba | 0.2 (0.1,0.5) |  |  |  |  |
| Abbreviations: CI- confidence interval; HH- households; N-number; n/a- not applicable; aOR- adjusted odds ratio; ref: reference  ^1^ Adjusted for sex of head of household, age of head of household, education of head of household, household size, presence of currently/recently pregnant woman, presence of child under five years old, residence, wealth quintile and region (not shown in Table)  ^2^ There was no variable for education level of head of household in the dataset. | | | | | | | | | | | | | |

**Additional Table S3. Among countries with over 10% of nets from the private sector: factors associated with a purchased net being used the previous night: region**

| **Angola 2015-16 DHS** | | **Madagascar 2016 MIS** | | **Malawi 2017 MIS** | | **Mali 2015 MIS** | | **Tanzania 2017 MIS** | | **Uganda 2016 DHS** | | **Zimbabwe 2015 DHS** | |
| --- | --- | --- | --- | --- | --- | --- | --- | --- | --- | --- | --- | --- | --- |
| **Odds of households owning a private sector net: aOR^1^ (95% CI)** | | | | | | | | | | | | | |
| Cabinda | ref | Analamanga | ref | Northern region | ref | Kayes | ref | Dodoma | ref | Kampala | ref | Manicaland | ref |
| Zaire | 0.5 (0.2,1.7) | Vakinankaratra | 1.2 (0.1,13.2) | Central region | 0.6 (0.4,0.8) | Koulikoro | -- | Arusha | 1.2 (0.4,4.2) | Central 1 | 0.9 (0.6 -1.3) | Mahonaland Central | 0.6 (0.2,2.1) |
| Uige | 0.3 (0.1,0.7) | Itasy | 1.4 (0.5,3.6) | Southern region | 0.8 (0.6,1.1) | Sikasso | -- | Kilimanjaro | 1.6 (0.5,6.0) | Central 2 | 0.9 (0.6,1.6) | Mashonaland East | 0.9 (0.4,2.3) |
| Luanda | 0.7 (0.3,1.7) | Bongolova | 0.6 (0.0,123.0) |  |  | Segou | -- | Tanga | 1.0 (0.4, 2.5) | Busoga | 0.9 (0.5,1.5) | Mashonaland West | 0.7 (0.3,2.0) |
| Cuanza Norte | 0.2 (0.1,0.5) | Haute Matsiatra | 0.7 (0.3,1.6) |  |  | Mopti | -- | Morogoro | 6.2 (2.2, 17.2) | Bukedi | 0.8 (0.4,1.4) | Matabeleland North | 0.7 (0.2,2.2) |
| Cuanza Sul | 0.2 (0.1,0.7) | Amoron I Mania | 0.7 (0.3,1.6) |  |  | Bamako | -- | Pwani | 4.1 (1.8, 9.4) | Bugishu | 1.1 (0.6,2.0) | Matabeleland South | 0.9 (0.4,2.4) |
| Malanje | 0.2 (0.1,0.7) | Vatovavy Fitovinany | 11.3 (2.3,56.9) |  |  |  |  | Dar Es Salaam | 2.3 (1.0,5.2) | Teso | 1.2 (0.7,2.0) | Midlands | 0.9 (0.3,2.3) |
| Lunda Norte | 0.8 (0.1,5.3) | Ihorombe | 0.8 (0.2,3.8) |  |  |  |  | Lindi | 2.3 (1.0,5.2) | Karamoja | 0.5 (0.3,1.1) | Masvingo | 0.6 (0.2,1.5) |
| Benguenla | 0.3 (0.1,5.3) | Atsimo Atsinanana | 1.8 (0.4,7.6) |  |  |  |  | Mtwara | 6.5 (2.6,16.1) | Lango | 0.7 (0.3,1.6) | Harare | 0.6 (0.3,1.4) |
| Huambo | 0.6 (0.2,1.8) | Atsinanana | 0.8 (0.3,2.2) |  |  |  |  | Ruvuma | 2.3 (0.90,5.8) | Acholi | 0.9 (0.5,1.5) | Bulawayo | 1.8 (0.9,3.6) |
| Bie | 0.3 (0.1,1.0) | Analanjirofo | 1.4 (0.6,3.5) |  |  |  |  | Iringa | 5.1 (0.7,38.9) | West Nile | 0.6 (0.3,0.9) |  |  |
| Moxico | 0.9 (0.3,2.3) | Alaotra Mangoro | 0.9 (0.3,2.7) |  |  |  |  | Mbeya | 0.9 (0.3,2.7) | Bunyoro | 0.8 (0.5,1.4) |  |  |
| Cuanda Cubango | 0.6 (0.2,1.5) | Boeny | 28.2 (3.6,219.2) |  |  |  |  | Singida | 0.7 (0.1,5.5) | Toor | 0.5 (0.2,1.2) |  |  |
| Namibe | 0.4 (0.1,1.6) | Sofia | 2.7 (0.8,9.5) |  |  |  |  | Tabora | 1.1 (0.5,2.5) | Ankole | 0.4 (0.3,0.8) |  |  |
| Huila | 0.2 (0.1,0.4) | Betsiboka | 1.8 (0.5,7.1) |  |  |  |  | Rukwa | 0.6 (0.3,1.5) | Kigezi | 0.5 (0.3,1.0) |  |  |
| Cunene | 0.1 (0.1,0.3) | Melaky | 1.1 (0.3,3.6) |  |  |  |  | Kigoma | 3.4 (1.1,11.1) |  |  |  |  |
| Lunda Sul | 0.2 (0.1,0.9) | Atsimo Andrefana | 0.8 (0.1,4.4) |  |  |  |  | Shinyanga | 2.8 (1.3,6.3) |  |  |  |  |
| Bengo | 0.2 (0.0,1.1) | Androy | 5.9 (0.8,43.9) |  |  |  |  | Kagera | 1.3 (0.5,3.9) |  |  |  |  |
|  |  | Anosy | 0.6 (0.2,1.6) |  |  |  |  | Mwanza | 4.8 (1.9,12.2) |  |  |  |  |
|  |  | Menabe | 5.1 (1.1,23.9) |  |  |  |  | Mara | 3.4 (1.5,7.6) |  |  |  |  |
|  |  | Diana | 5.6 (1.6,19.9) |  |  |  |  | Manyara | 3.4 (0.8,15.1) |  |  |  |  |
|  |  | Sava | 2.7 (1.1,7.1) |  |  |  |  | Njombe | 0.7 (0.2,3.0) |  |  |  |  |
|  |  |  |  |  |  |  |  | Katavi | 2.4 (0.9,6.7) |  |  |  |  |
|  |  |  |  |  |  |  |  | Simiyu | 1.8 (0.6,4.9) |  |  |  |  |
|  |  |  |  |  |  |  |  | Geita | 1.9 (0.6,5.5) |  |  |  |  |
|  |  |  |  |  |  |  |  | Songwe | 1.3 (0.2,7.3) |  |  |  |  |
|  |  |  |  |  |  |  |  | Kaskazihi Unguja | 1.4 (0.7,2.6) |  |  |  |  |
|  |  |  |  |  |  |  |  | Kusini Unguja | 0.4 (0.1,1.7) |  |  |  |  |
|  |  |  |  |  |  |  |  | Mjini Magharibi | 4.8 (1.6,14.4) |  |  |  |  |
|  |  |  |  |  |  |  |  | Kaskazini Pemba | -- |  |  |  |  |
|  |  |  |  |  |  |  |  | Kusini Pemba | 4.6 (0.8,24.9) |  |  |  |  |
| Abbreviations: CI: confidence interval; HH- households; N-number; n/a: not applicable; aOR: adjusted odds ratio; ref: reference  ^1^ Adjusted for sex of head of household, age of head of household, education of head of household, household size, presence of currently/recently pregnant woman, presence of child under five years old, residence, wealth quintile and region (not shown in Table)  ^2^ There was no variable for education level of head of household in the dataset. | | | | | | | | | | | | | |
